# Novel phenotypes of coronavirus disease: a temperature-based trajectory model

**DOI:** 10.1186/s13613-021-00907-4

**Published:** 2021-08-03

**Authors:** Yanfei Shen, Dechang Chen, Xinmei Huang, Guolong Cai, Qianghong Xu, Caibao Hu, Jing Yan, Jiao Liu

**Affiliations:** 1 Department of Intensive Care, Zhejiang Hospital, Hangzhou, Zhejiang China; 2 Department of Intensive Care, Ruijin Hospital, Shanghai Jiao Tong University School of Medicine, Shanghai, China; 3 Department of Internal Medicine, The Second Affiliated Hospital, Zhejiang University School of Medicine, Hangzhou, Zhejiang China; 4 Department of Critical Care Medicine, Ruijin Hospital, Shanghai Jiao Tong University School of Medicine, No. 197 Ruijin 2nd Road, Shanghai, 200025 China

**Keywords:** COVID-19, Temperature, Mortality, Inflammatory response, Corticosteroids

## Abstract

**Background:**

Coronavirus disease has heterogeneous clinical features; however, the reasons for the heterogeneity are poorly understood. This study aimed to identify clinical phenotypes according to patients’ temperature trajectory.

**Method:**

A retrospective review was conducted in five tertiary hospitals in Hubei Province from November 2019 to March 2020. We explored potential temperature-based trajectory phenotypes and assessed patients’ clinical outcomes, inflammatory response, and response to immunotherapy according to phenotypes.

**Results:**

A total of 1580 patients were included. Four temperature-based trajectory phenotypes were identified: normothermic (Phenotype 1); fever, rapid defervescence (Phenotype 2); gradual fever onset (Phenotype 3); and fever, slow defervescence (Phenotype 4). Compared with Phenotypes 1 and 2, Phenotypes 3 and 4 had a significantly higher C-reactive protein level and neutrophil count and a significantly lower lymphocyte count. After adjusting for confounders, Phenotypes 3 and 4 had higher in-hospital mortality (adjusted odds ratio and 95% confidence interval 2.1, 1.1–4.0; and 3.3, 1.4–8.2, respectively), while Phenotype 2 had similar mortality, compared with Phenotype 1. Corticosteroid use was associated with significantly higher in-hospital mortality in Phenotypes 1 and 2, but not in Phenotypes 3 or 4 (*p* for interaction < 0.01). A similar trend was observed for gamma-globulin.

**Conclusions:**

Patients with different temperature-trajectory phenotypes had different inflammatory responses, clinical outcomes, and responses to corticosteroid therapy.

**Supplementary Information:**

The online version contains supplementary material available at 10.1186/s13613-021-00907-4.

## Background

Coronavirus disease (COVID-19) is a global health threat that has caused more than one million deaths worldwide. Concerted efforts have been made to evaluate effective therapies, such as use of corticosteroids [1, 2] and statins [3, 4]. However, the effectiveness of various therapies is unclear.

Evidence has indicated that COVID-19 is a heterogeneous disease [5, 6] characterized by different inflammatory responses. For instance, the clinical course of most COVID-19 patients is relatively mild or asymptomatic, while some patients develop severe clinical complications [7]. Despite the heterogeneity within COVID-19 cases, most clinical trials have focused on a one-size-fits-all treatment approach, which may partly explain the inconsistencies in the results between studies. Early identification of different clinical phenotypes may contribute to improving medical interventions and prognosis [8, 9].

Fever occurs in 88.7% of COVID-19 patients at the time of hospital admission [10, 11]. Thermoregulation disorder (i.e., hypothermia or fever) is associated with different clinical outcomes [12–16] and immunological status [17–19]. However, one common limitation of previous studies is that only static temperature values were used, which failed to reflect the dynamic body temperature change in clinical practice.

Thus, using a temperature-based trajectory model [20], we conducted a multicenter cohort study to explore the effects of potential body temperature-based trajectories using longitudinal temperature monitoring data. The purpose of this study was to investigate the heterogeneity between different trajectories from the perspective of clinical outcomes, inflammatory markers, and response to immunotherapy.

## Material and methods

### Study participants

The study was performed in five tertiary hospitals in Hubei Province in China: Union Jiangbei Hospital, Wuhan No.9 Hospital, Wuhan No.4 Hospital, Wuhan Jinyintan Hospital, and Huangshi Central Hospital. All patients admitted to these hospitals from November 20, 2019 to March 20, 2020 were screened. Patients who met the following inclusion criteria were included: (1) a diagnosis of COVID-19 according to the World Health Organization guidelines; and (2) age over 18 years. Patients without records of their body temperature were excluded. This study was approved by the ethics committees of each of the five hospitals. The requirement for patient informed consent was waived owing to the retrospective study design.

### Data collection

We collected data from electronic medical records of each center, including those on demographics, comorbidities, laboratory results, transfer, disease severity, use of immunotherapy (such as corticosteroids, gamma-globulin, and interferon), and clinical outcomes. Data were extracted on each patient’s maximum daily body temperature during the first 5 days after hospital admission and used for trajectory modeling. Authors Q.X. and C.H. were responsible for data verification.

### Outcome definition

This study aimed to investigate the heterogeneous features of temperature-trajectory phenotypes from three perspectives: the clinical outcome, inflammatory response, and response to immunotherapy. In-hospital mortality was the primary clinical outcome. Other clinical outcomes such as intubation rate, acute respiratory distress syndrome (ARDS), intensive care unit (ICU) admission, and length of hospitalization and ICU stay were secondary outcomes. Lymphocyte count, neutrophil count, and C-reactive protein (CRP) level were used as markers of the immune response. Finally, we evaluated the responses to corticosteroids, gamma-globulin, and interferon therapies as measures of the response to immunotherapy for each temperature-trajectory phenotype.

### Management of missing data

In this study, most of the continuous variables had less than 5% of values missing. Missing values were replaced by the mean or median value in the analysis. Variables with more than 20% of values missing were not imputed. Four missing values of sex were replaced by the default value (zero, regarded as male).

### Sensitivity analysis

To test the robustness of our findings, we also constructed another trajectory model based on the maximum temperature records within 3 days after admission. The consistency of the discriminability of these two models was investigated.

### Statistical analysis

Categorical variables are summarized as proportions, and the difference was tested using the Chi-square or Fisher’s exact tests. Continuous variables are summarized as the mean ± standard deviation or the median and interquartile range, according to the distribution, and were compared using *t* tests or Wilcoxon rank-sum tests.

Group-based trajectory modeling is a growth mixture modeling method that is used to map the progression of symptoms and assess heterogeneous responses to clinical interventions [20]. In this study, the maximum daily body temperature within 5 days after hospital admission was used to build the trajectory models, identifying patients with a similar temperature trajectory. The “traj” statistical application in Stata software was used to fit a censored normal model. The development trajectories of body temperature were explored when they were divided into one, two, three, and four trajectories. Evaluation of model fitting was based on the following standards: (1) the Bayesian information criterion was used to determine the number of trajectories; (2) the log Bayes factor was used to determine whether the complex or the simple model should be used; and (3) the average posterior probability was used to evaluate the posterior probability that each individual was assigned to the corresponding temperature-trajectory phenotype, using an acceptable value of 0.7.

The multivariable logistic regression model was built as follows: variables with *p* < 0.2 in the univariate comparisons were included in the initial model and a backward stepwise approach was applied to exclude confounders with *p* > 0.10; however, variables with a great impact on the coefficient of temperature-trajectory phenotypes (> 10%) were retained in the final model. The variance inflation factor method was used to test multicollinearity, and a bootstrap technique using 1000 resamples was used to test model stability. Stratified analyses of the predicted marginal effects of immunotherapy were also performed according to the temperature-trajectory phenotype. Two-tailed *p* values < 0.05 were considered statistically significant. All statistical analyses were performed using Stata 14.0 (College Station, TX, USA).

## Results

### Baseline characteristics of survivors and non-survivors

A total of 1580 patients were included in this study, with an overall mortality rate of 16.4% (Table 1). Their mean age was 57 years, and 53.1% were male. Compared with the survivors, the non-survivors had a significantly stronger inflammatory response, such as higher white blood cell and neutrophil counts, higher CRP levels, and lower lymphocyte counts. The non-survivors were more likely to receive immunotherapy, such as the use of corticosteroids, gamma-globulin, and interferon.

**Table 1:** Comparison of baseline characteristics between survivors and non-survivors

Demographics	Survivors (*n* = 1357)	Non-survivors (*n* = 223)	All patients (*n* = 1580)	*p*
Age (years)	56.2 ± 13.9	67.1 ± 12.4	57.7 ± 14.2	< 0.001
Male [*n* (%)]	703 (51.8)	136 (60.9)	839 (53.1)	0.011
Comorbidities				
Hypertension [*n* (%)]	322 (23.7)	75 (33.6)	397 (25.1)	0.002
Diabetes mellitus [*n* (%)]	141 (10.3)	43 (19.2)	184 (11.6)	< 0.001
Chronic heart diseases [*n* (%)]	77 (5.6)	27 (12.1)	104 (6.5)	< 0.001
COPD [*n* (%)]	15 (1.1)	11 (4.9)	26 (1.6)	< 0.001
Chronic renal diseases [*n* (%)]	27 (2.0)	7 (3.1)	34 (2.1)	0.273
Malignant tumor [*n* (%)]	31 (2.3)	14 (6.2)	45 (2.8)	< 0.001
Laboratory and disease severity indexes				
Initial white blood cell count (10^9/L)	6.6 ± 3.2	10.2 ± 5.2	7.1 ± 3.7	< 0.001
Maximum white blood cell count (10^9/L)	7.8 ± 3.6	16.5 ± 8.8	9.0 ± 5.6	< 0.001
Initial neutrophil count (10^9/L)	4.6 ± 2.6	8.3 ± 4.8	5.1 ± 3.3	< 0.001
Maximum neutrophil count (10^9/L)	5.7 ± 3.3	14.1 ± 8.0	6.9 ± 5.2	< 0.001
Initial lymphocyte cell count (10^9/L)	1.2 ± 0.6	0.8 ± 0.6	1.1 ± 0.6	< 0.001
Minimum lymphocyte cell count (10^9/L)	1.0 ± 0.6	0.5 ± 0.4	0.9 ± 0.6	< 0.001
Initial hemoglobin level (g/dl)	122.6 ± 16.7	119.9 ± 19.1	122.2 ± 17.1	0.025
Initial platelet count (10 /L)	224 ± 89	166 ± 73	216 ± 89	< 0.001
Initial albumin level (g/L)	33.9 ± 4.3	30.4 ± 4.2	33.4 ± 4.5	< 0.001
Initial serum creatinine (mmol/l)	72.1 ± 33.1	96.3 ± 78.6	75.5 ± 43.3	< 0.001
PaO2/FiO2	283 ± 128	170 ± 146	266 ± 137	< 0.001
Initial CRP level (mmol/L)	29 ± 35 (n = 30)	84 ± 49 (n = 43)	36 ± 41 (n = 343)	< 0.001
Maximum CRP level (mmol/L)	38 ± 41 (n = 30)	118 ± 46 (n = 43)	48 ± 49 (n = 343)	< 0.001
SOFA score on admission [median (IQR)]	1 (0–2)	3 (1–4)	1 (0–2)	< 0.001
Maximum SOFA score [median (IQR)]	1 (0–2)	6 (4–9)	1 (0–2)	< 0.001
Study center				0.011
Union Jiangbei Hospital	160 (11.7)	35 (15.6)	195 (12.3)	
Wuhan No.9 Hospital	138 (10.1)	25 (11.2)	163 (10.3)	
Wuhan No.4 Hospital	42 (3.1)	11 (4.9)	53 (3.3)	
Wuhan Jinyintan Hospital	1003 (73.9)	145 (65.0)	1148 (72.6)	
Huangshi central Hospital	14 (1.0)	7 (3.1)	21 (1.3)	
Immunotherapy				
Corticosteroid therapy [n (%)]	300 (22.1)	117 (52.4)	417 (26.3)	< 0.001
Interferon therapy [n (%)]	198 (14.5)	34 (15.2)	232 (13.8)	0.798
Gamma-globulin therapy [n (%)]	270 (19.8)	88 (39.4)	358 (21.3)	< 0.001
Clinical outcomes				
ARDS [*n* (%)]	180 (13.2)	197 (88.3)	377 (23.8)	< 0.001
Mechanical ventilation [*n* (%)]	26 (1.9)	78 (34.9)	104 (6.5)	< 0.001
Duration of mechanical ventilation (days)	10.3 ± 3.7	5.6 ± 4.6	6.7 ± 4.8	< 0.001
Hospital length of stay (days)	13.5 ± 7.1	10.5 ± 7.0	13.0 ± 7.1	< 0.001
ICU admission [*n* (%)]	164 (12.1)	109 (48.8)	273 (17.2)	< 0.001
ICU length of stay (days)	10.4 ± 10.3	8.3 ± 7.3	9.6 ± 9.3	0.064

*COPD* chronic obstructive pulmonary disease, *PaO*_*2*_*/FiO*_*2*_ ratio of partial pressure of arterial oxygen to fraction of inspired oxygen, *SOFA* Sequential Organ Failure Assessment, *ARDS* acute respiratory distress syndrome, *ICU* intensive care unit

### Selection of temperature-based trajectory models

**Fig. 1:**
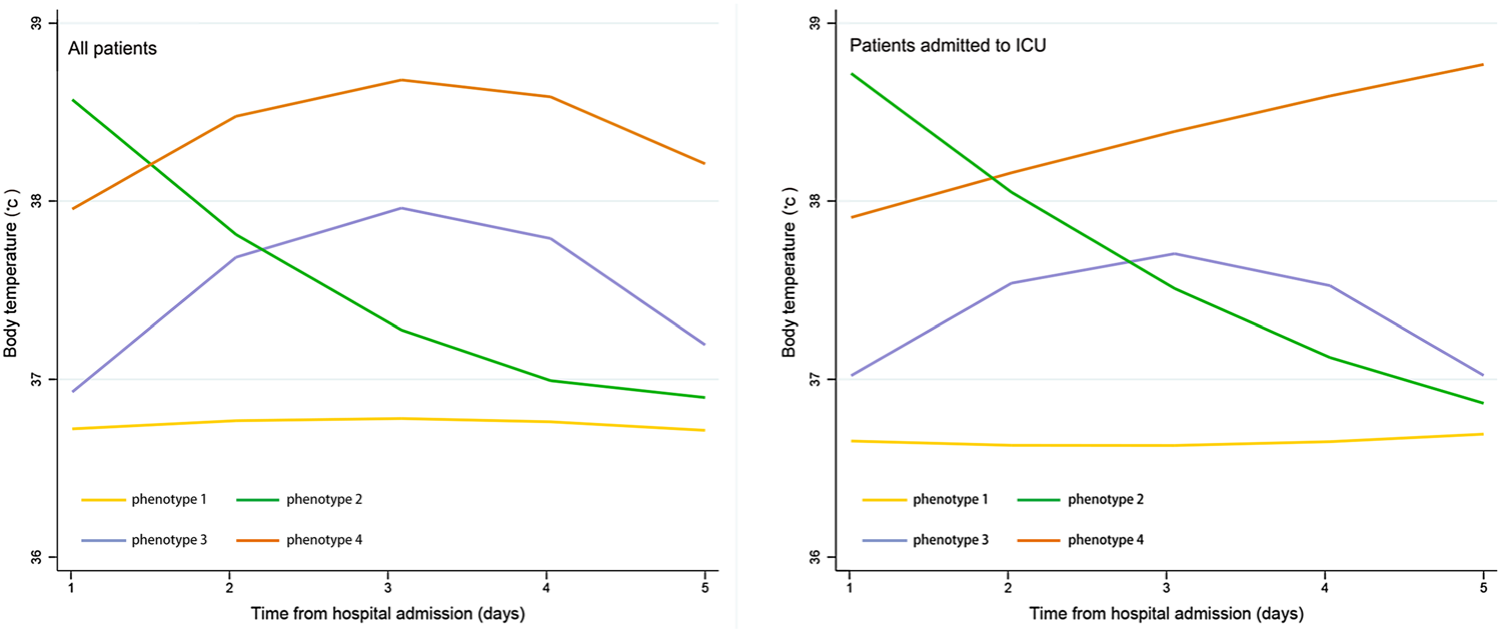
Temperature-trajectory phenotypes in COVID-19 patients. Phenotype 1 (normothermic: yellow line)—patients with normal body temperature; Phenotype 2 (fever, rapid defervescence: green line)—patients with fever on admission but rapid defervescence; Phenotype 3 (gradual fever onset: purple line)—patients had normal temperature on admission but had fever later; Phenotype 4 (fever, slow defervescence: orange line)—patients with fever on admission and slow defervescence. *COVID-19* coronavirus disease

The phenotype selection process of the trajectory model is shown in Table S1 [see Additional file 1]. Based on the Bayesian information criterion and log Bayes factor, a trajectory model with four phenotypes was adopted (shown in Fig. 1): Phenotype 1 (normothermic), patients with a normal body temperature (*n* = 1217); Phenotype 2 (fever, rapid defervescence), patients with fever on admission but rapid defervescence (*n* = 189); Phenotype 3 (gradual fever onset), patients with a normal temperature on admission who developed a fever later (*n* = 122); and Phenotype 4 (fever, slow defervescence), patients with fever on admission and slow defervescence (*n* = 52). Each of the four temperature trajectories was found to be the product of a unique quadratic function describing temperature as a function of time. Temperature trajectories were also modeled in patients admitted to the ICU. The overall trajectories remained stable.

### Body temperature characteristics within phenotypes

Most of the patients with Phenotype 1 had normal body temperature records, and only 3% of these patients still had fever (temperature > 38 °C) on day 3 (Table 2). The maximum body temperature of patients with Phenotype 2 was similar to that of patients with Phenotypes 3 and 4 (38.7, 38.5, and 39.3 °C, respectively). However, compared with those in Phenotypes 3 and 4, the time to maximum body temperature was significantly shorter in patients with Phenotype 2 (3.0 ± 0.9, 3.1 ± 1.0, and 1.4 ± 0.7, respectively, *p* < 0.001); the proportion of patients with persistent fever (body temperature on day 3 > body temperature on day 1) was significantly lower (15/189 [7.9%], 115/122 [94.2%], and 36/52 [69.2%], *p* < 0.001) in Phenotype 3. The detailed body temperature profiles are shown in Table 2.

**Table 2:** Comparison of body temperature within the four temperature-trajectory phenotypes

	Phenotype-1 Normothermic	Phenotype-2 Fever, fast resolvers	Phenotype-3 Gradual fever onset	Phenotype-4 Fever, slow resolvers	*p*
BT on admission (°C)	36.7 ± 0.4	38.5 ± 0.5	36.8 ± 0.4	37.9 ± 0.8	< 0.001
Maximum BT (°C)	37.1 ± 0.4	38.7 ± 0.5	38.5 ± 0.4	39.3 ± 0.5	< 0.001
Minimum BT (°C)	36.4 ± 0.1	36.6 ± 0.4	36.6 ± 0.3	37.3 ± 0.6	< 0.001
Mean BT (°C)	36.7 ± 0.2	37.5 ± 0.3	37.5 ± 0.2	38.4 ± 0.3	< 0.001
Maximum BT difference (°C)	0.7 ± 0.4	2.0 ± 0.7	1.8 ± 0.6	2.0 ± 0.9	< 0.001
Time to maximum BT (Days)	3.1 ± 1.4	1.4 ± 0.7	3.0 ± 0.9	3.1 ± 1.0	< 0.001
BT on day3 > BT on day 1, *n* (%)	595/1217 (48.9)	15/189 (7.9)	115/122 (94.2)	36/52 (69.2)	< 0.001
BT on day3 > 38 °C, *n* (%)	37/1217 (3.0)	32/189 (16.9)	57/122 (46.7)	45/52 (86.0)	< 0.001
Fever (BT > 38 °C), *n* (%)	46/5812 (0.8)	264/906 (29.1)	152/588 (25.8)	173/245 (70.6)	< 0.001
BT trends within 5 days					
BT on day 1 (°C)	36.7 ± 0.4	38.5 ± 0.5	36.8 ± 0.4	37.9 ± 0.8	< 0.001
BT on day 2 (°C)	36.8 ± 0.4	37.8 ± 0.8	37.8 ± 0.7	38.4 ± 0.9	< 0.001
BT on day 3 (°C)	36.7 ± 0.4	37.2 ± 0.7	38.0 ± 0.7	38.8 ± 0.9	< 0.001
BT on day 4 (°C)	36.7 ± 0.3	37.0 ± 0.4	37.6 ± 0.6	38.4 ± 0.8	< 0.001
BT on day 5 (°C)	36.7 ± 0.3	36.8 ± 0.5	37.3 ± 0.6	38.3 ± 0.9	< 0.001

*BT* body temperature

### Inflammatory response according to phenotypes

**Fig. 2:**
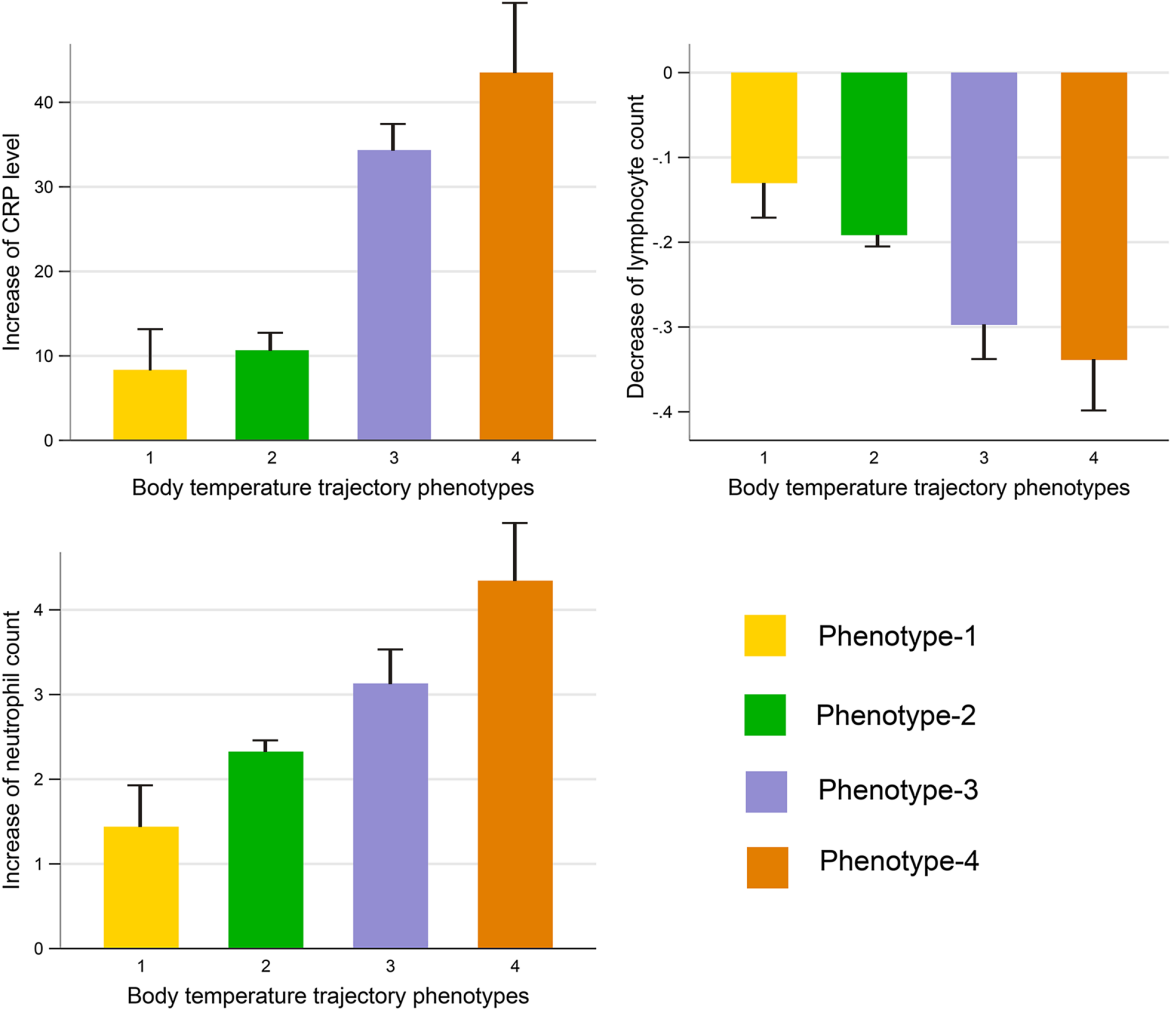
Comparisons of inflammatory marker levels according to the temperature-trajectory phenotype. Compared with those in Phenotypes 1 and 2 (normothermic or fever with rapid defervescence), the change in the CRP level is significantly higher (*p* < 0.001) and the change in the lymphocyte count is significantly lower (*p* < 0.001) in Phenotypes 3 and 4. The maximum SOFA score showed a stepwise increase from Phenotype 1–4 (*p* < 0.001). The vertical line above each bar represents the standard error of mean. *CRP* C-reactive protein, *SOFA* Sequential Organ Failure Assessment

The changes in serum CRP levels and neutrophil and lymphocyte counts, which were used as indicators of the inflammatory response, are shown in Fig. 2 and Table 3. Compared with those in Phenotype 1 (normothermic) and Phenotype 2 (fever, rapid defervescence), the CRP level and the neutrophil count significantly increased (*p* < 0.001), while the lymphocyte count significantly decreased (*p* < 0.001) in Phenotype 3 (gradual fever onset) and Phenotype 4 (fever, slow defervescence). Other detailed comparisons are provided in Table 3.

**Table 3:** Comparison of patient characteristics, therapy, and outcome within the four temperature-trajectory phenotypes

Variables	Phenotype-1 Normothermic (*n* = 1217)	Phenotype-2 Fever, fast resolvers (*n* = 189)	Phenotype-3 Gradual fever onset (*n* = 122)	Phenotype-4 Fever, slow resolvers (*n* = 52)	*p*
Demographic data					
Age [*n* (%)]	57.7 ± 14.6	58.1 ± 13.4	57.7 ± 12.2	55.2 ± 13.0	0.639
Male [*n* (%)]	603 (49.5)	117 (61.9)	74 (60.6)	44 (84.6)	< 0.001
Hypertension [*n* (%)]	314 (25.8)	37 (19.5)	34 (27.8)	12 (23.0)	0.262
Diabetes mellitus [*n* (%)]	145 (11.9)	20 (10.5)	13 (10.6)	6 (11.5)	0.938
Chronic heart diseases [*n* (%)]	90 (7.3)	7 (3.7)	6 (4.9)	1 (2.0)	0.100
Severity of COVID-19					< 0.001
Mild [*n* (%)]	846 (69.5)	105 (55.5)	64 (52.4)	26 (50.0)	
Severe [*n* (%)]	251 (20.6)	50 (26.4)	27 (22.1)	12 (21.4)	
Critical [*n* (%)]	120 (9.8)	34 (17.9)	31 (25.4)	14 (26.9)	
Inflammatory markers					
Initial neutrophil count (10^9/L)	5.0 ± 3.2	5.8 ± 3.5	5.5 ± 3.7	4.7 ± 3.5	< 0.001
Maximum neutrophil count (10^9/L)	6.4 ± 4.9	8.1 ± 5.7	8.7 ± 6.1	9.1 ± 3.9	< 0.001
Change of neutrophil count (10^9/L)	1.4 ± 3.4	2.3 ± 4.4	3.1 ± 4.6	4.3 ± 5.2	< 0.001
Initial lymphocyte count (10^9/L)	1.2 ± 0.6	1.0 ± 0.6	1.0 ± 0.5	0.9 ± 0.5	< 0.001
Minimum lymphocyte count (10^9/L)	1.0 ± 0.6	0.8 ± 0.5	0.7 ± 0.4	0.6 ± 0.3	< 0.001
Change of lymphocyte count (10^9/L)	− 0.13 ± 0.30	− 0.19 ± 0.41	− 0.30 ± 0.40	− 0.34 ± 0.44	< 0.001
Initial CRP level (mmol/L)	30 ± 39 (*n* = 250)	54 ± 42 (*n* = 47)	40 ± 44 (*n* = 33)	62 ± 52 (*n* = 13)	< 0.001
Maximum CRP level (mmol/L)	39 ± 45	64 ± 43	75 ± 57	105 ± 53	< 0.001
Change of CRP level (mmol/L)	8 ± 24	10 ± 24	34 ± 37	43 ± 41	< 0.001
Initial SOFA score	1 (0–2)	2 (1–2)	1 (0–1)	2 (1–2.5)	< 0.001
Maximum SOFA score	1 (0–2)	2 (1–3)	2 (1–4)	2 (1–4)	< 0.001
Immunotherapy					
Corticosteroid therapy [*n* (%)]	314 (25.8)	94 (49.7)	49 (40.1)	32 (61.5)	< 0.001
Gamma-globulin therapy [*n* (%)]	257 (21.1)	70 (37.0)	54 (44.2)	25 (48.0)	< 0.001
Interferon therapy [*n* (%)]	187 (15.3)	22 (11.6)	18 (14.7)	5 (9.6)	0.405
Clinical outcomes					
In-hospital death [*n* (%)]	138 (11.3)	39 (20.6)	31 (25.4)	15 (28.8)	< 0.001
ARDS [*n* (%)]	249 (20.4)	65 (34.3)	44 (36.0)	19 (36.5)	< 0.001
Intubation [*n* (%)]	61 (5.0)	24 (12.7)	11 (9.0)	8 (15.4)	< 0.001
Duration of mechanical ventilation (days)	6.7 ± 5.2	7.1 ± 5.4	5.9 ± 4.1	6.2 ± 5.5	0.905
Hospital length of stay (days)	12.4 ± 6.8	14.7 ± 8.2	15.2 ± 7.3	16.8 ± 7.6	< 0.001

*CRP* C-reactive protein, *SOFA* Sequential Organ Failure Assessment, *ARDS* acute respiratory distress syndrome

### Clinical outcomes according to phenotypes

The comparisons of clinical outcomes between these four phenotypes are shown in Table 3. The crude in-hospital mortality, ARDS incidence, and hospital length of stay gradually increased from Phenotype 1 (normothermic) to Phenotype 4 (fever, slow defervescence). In the multivariable logistic regression model (Table 4), the odds ratio (OR) for mortality relative to Phenotype 1 (normothermic) was significantly higher in patients with Phenotype 3 (gradual fever onset, OR: 2.1, 95% confidence interval [CI]: 1.1–4.0) and Phenotype 4 (fever, slow defervescence, OR: 3.3, 95% CI: 1.4–8.2), but not Phenotype 2 (fever, rapid defervescence, OR: 1.2, 95% CI: 0.7–2.2). This trend remained stable when tested using bootstrapping with 1,000 resamples.

**Table 4:** Association between in-hospital death and temperature-trajectory phenotype in logistic regression models

	Model 1		Model 2		Model 3	
	Crude OR (95% CI)	*p*	Multivariable logistic model aOR (95% CI)	*p*	Multivariable logistic model with 1000 bootstraps, aOR (95% CI)	*p*
Normothermic	Ref.	–	Ref.	–	Ref.	–
Fever, fast resolvers	2.0 (1.3–3.0)	< 0.001	1.2 (0.7–2.2)	0.377	1.2 (0.6–2.2)	0.364
Gradual fever onset	2.6 (1.7–4.1)	< 0.001	2.1 (1.1–4.0)	0.021	2.1 (1.1–3.9)	0.013
Fever, slow resolvers	3.2 (1.6–3.9)	< 0.001	3.3 (1.4–8.2)	0.005	3.4 (1.3–8.5)	0.007
Age > 65 (years)			3.4 (2.2–5.1)	< 0.001	3.4 (2.1–5.3)	< 0.001
Vasopressor use			3.1 (2.0–4.7)	< 0.001	3.0 (1.9–4.8)	< 0.001
Maximum creatinine level			1.01 (1.00–1.01)	< 0.001	1.01 (1.00–1.01)	0.010
Maximum WBC count			1.2 (1.1–1.3)	< 0.001	1.2 (1.2–1.3)	< 0.001
APACHE II score			1.2 (1.1–1.3)	< 0.001	1.3 (1.1–1.4)	< 0.001

Three logistic models were used to evaluate the association between in-hospital death and four body temperature trajectory groups. Compared to the normothermic group, there was a trend toward increasing risk of in-hospital death from fever, fast resolvers to fever, and slow resolvers. Bootstrapping (1000 resamples) were used for calculating 95% CI in Model 3 and the results remained stable

*aOR* adjusted odds ratio, *APACHE II* acute physiology and chronic health evaluation; *CI* confidence interval, *WBC* white blood cell

### Response to immunotherapy according to phenotypes

**Fig. 3:**
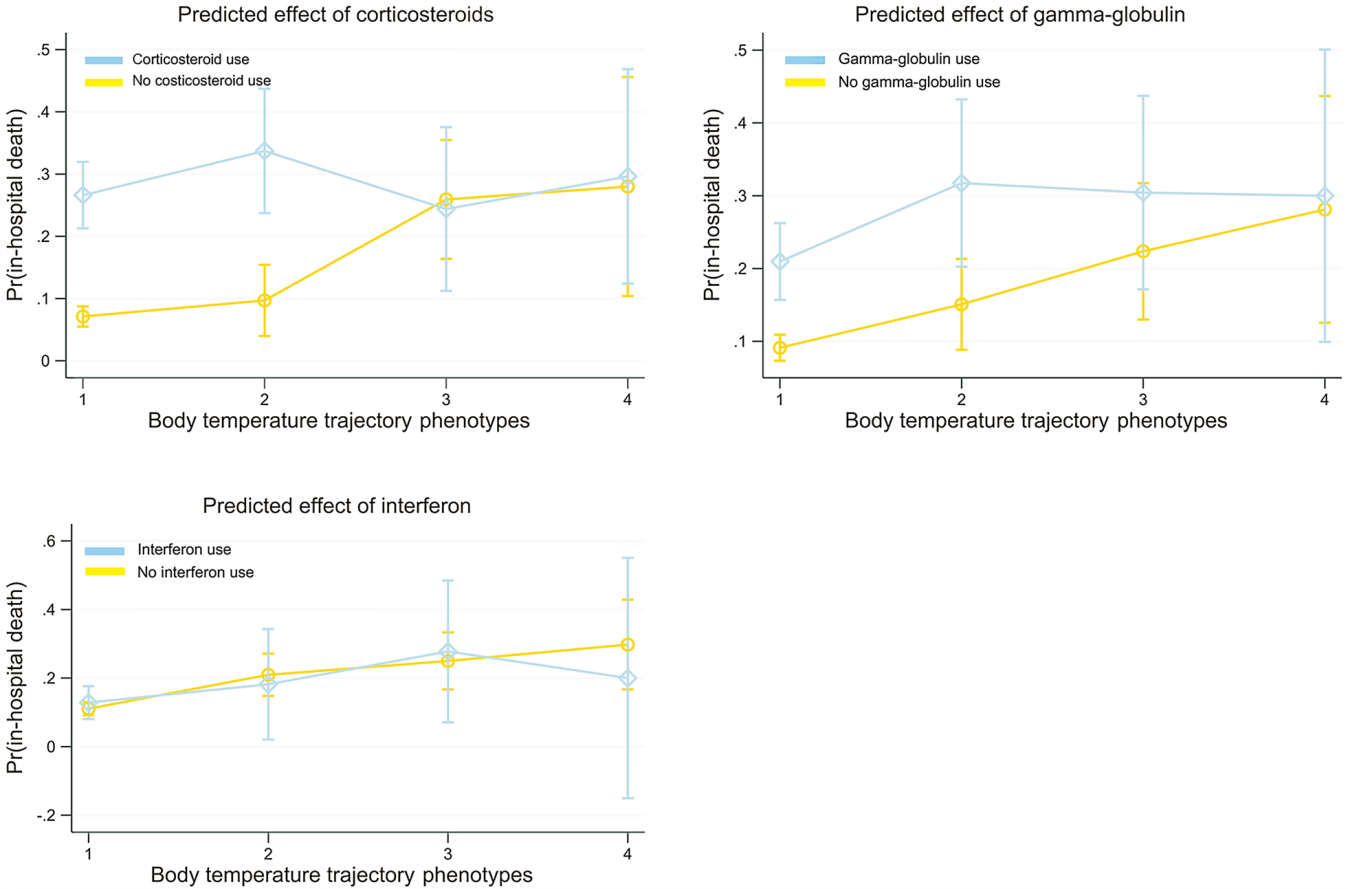
The predicted marginal effect of immunotherapy in each temperature-trajectory phenotype. Corticosteroid use was associated with significantly increased in-hospital mortality in Phenotypes 1 and 2, while this association was not significant in Phenotypes 3 and 4 (*p* for interaction < 0.01). A similar trend was observed for gamma-globulin; however, the difference in effect size between the four temperature-trajectory phenotypes was not significant (*p* for interaction > 0.05). The effectiveness of interferon therapy was consistent for all four phenotypes

Data on prescriptions of corticosteroids, gamma-globulin, and interferon in the 5 days after hospital admission were extracted and defined as immunotherapy. The differential response to immunotherapy according to the temperature-trajectory phenotype and the predicted marginal effects are shown in Fig. 3. Corticosteroid use was significantly associated with increased hospital mortality in patients with Phenotype 1 (normothermic) and Phenotype 2 (fever, rapid defervescence) (OR: 4.7, 95% CI: 3.2–6.8; and OR: 4.7, 95% CI: 2.1–10.4, respectively), while this association was not significant in patients with Phenotypes 3 and 4 (*p* for interaction < 0.01). A similar trend was observed for gamma-globulin; however, the difference in the effect size between the four phenotypes was not significant (*p* for interaction > 0.05). The effectiveness of interferon therapy was consistent across all four phenotypes.

### Sensitivity analysis

Another trajectory model was built based on temperature records within 3 days after hospital admission (Additional file 1: Fig. S1). This new model classified 92% of the patients into the same groups as the original model, confirming that the classification using temperature-based trajectory model was stable. Other sensitivity analyses were conducted considering patients without corticosteroid use within 5 days after hospital admission (Additional file 1: Fig. S2) and those with a hospital length of stay of more than 5 days (Additional file 1: Fig. S3).

## Discussion

The study had three major findings. First, we identified four different temperature-trajectory phenotypes in patients with COVID-19 based on their daily maximum temperature. Second, the inflammatory response, the ARDS incidence rate, and the mortality rate differed according to the temperature-trajectory phenotype. Third, the response to immunotherapy differed significantly according to the temperature-trajectory phenotype, which suggests that there is an interaction between immunotherapy and inflammatory response in patients with COVID-19.

A dysregulated inflammatory response resulting from severe acute respiratory syndrome coronavirus-2 (SARS-CoV-2) infection plays a critical role in the pathogeneses of COVID-19 [21, 22]. There is increasing evidence that COVID-19 is a heterogeneous syndrome [5, 6, 23]. For example, the clinical syndrome varies greatly from asymptomatic or mildly symptomatic to ARDS and death [5, 7]. In addition, inconsistencies in the results of immunotherapy trials, such as the use of corticosteroids [1, 2] and statins [3, 4], suggest that COVID-19 is not a homogeneous disease. Therefore, identifying COVID-19 patients with homogeneous characteristics and minimizing the heterogeneity bias in clinical trials are critical and require urgent attention. However, research on the role of the disease phenotype remains limited.

Abnormal body temperature is one of the common symptoms of infectious diseases. In a descriptive study including 1,099 COVID-19 cases, 88.7% of the patients had fever on admission [11]. Many studies have assessed the prognostic and immunological significance of body temperature in various diseases [12–19], including sepsis and ARDS. However, the conclusions are inconsistent. One common limitation is that only static temperature values were used in most of the studies. The lack of consideration of the longitudinal dynamic temperature change may be an important reason for inconsistent findings between studies.

Aiming to overcome this limitation, a phenotype-based trajectory model was applied to fit the symptom progression [20]. In previous studies of sepsis [24, 25], Bhavani et al. constructed four trajectories based on longitudinal temperature data in sepsis and found that both the inflammatory markers and mortality rate were significantly different. In a recent study of COVID-19 [26], the authors identified four temperature trajectories, each presenting with a distinct prognosis. This research appears similar to our study. However, several differences should be noted. First, in Bhavani et al.’s study [24–26], the trajectories were constructed based on all temperature records, which reduced the temperature feature of each trajectory. For example, in patients with intermittent fever, the temperature trajectory would be lowered to fit all temperature records (both normal and elevated temperature values). This may be the reason for the small temperature difference between the four temperature trajectories in Bhavani et al.’s [26] study. Establishing a temperature trajectory based on all temperature records may reduce the temperature features of each phenotype, increase the difficulty in identification, and limit the clinical application. In the current study, the temperature-trajectory phenotypes were constructed based on maximum daily temperature, which presented with more significant temperature features. Bhavani et al. [26] reported that, compared with a temperature trajectory of a rapidly decreasing fever, temperature trajectories of fever, normothermia, and hypothermia were associated with a higher mortality. However, we found that compared with those in normothermic patients, the crude estimates of clinical outcomes such as the incidence of ARDS, intubation rate, mortality rate, and ICU admission rate were significantly higher in patients with fever. After adjusting for confounders, the temperature-trajectory phenotypes of patients with fever of gradual onset or fever with slow defervescence remained associated with increased mortality, while the phenotype of fever with rapid defervescence did not. In addition, we were also concerned that the temperature trajectories may be affected by corticosteroid use, hospital discharge, or death within 5 days of admission. However, in the sensitivity analyses excluding these patients, the results remained stable.

The exact mechanism cannot be inferred from our study owing to the retrospective design. However, we noted that the CRP level and neutrophil count increased and the lymphocyte count decreased stepwise from Phenotypes 1 to 4. In COVID-19, both the lymphocyte count and the CRP level have been reported to be inflammatory markers for poor outcomes [27–30]. Therefore, the high CRP level and the low lymphocyte count in Phenotypes 3 and 4 may indicate relatively strong inflammatory response, which partly account for the high mortality in these groups.

In addition, studies have shown that corticosteroids [1] and gamma-globulin [31, 32] therapies have an anti-inflammatory effect in many conditions. As a non-specific immunomodulator, gamma-globulin can affect lymphocyte differentiation, inhibit cytokine production, and suppress the inflammatory response [33, 34]. However, evidence of the effectiveness of gamma-globulin in COVID-19 is inconsistent. For example, one study [35] including 37 severe COVID-19 patients found that a combination of methylprednisolone and immunoglobulin could improve the prognosis of COVID-19 patients, while another study [36] found that immunoglobulin therapy did not improve in-hospital mortality rates or the need for mechanical ventilation in patients with severe COVID-19. Similarly, the results of different studies of corticosteroid use in COVID-19 showed inconsistent results. Some studies [1, 37] found that corticosteroid use was effective in patients with COVID-19, while other studies [2, 38] found that corticosteroid use was ineffective. In the current study, the effectiveness of corticosteroids and gamma-globulin therapy differed significantly between phenotypes with low (Phenotypes 1 and 2) and high (Phenotypes 3 and 4) inflammatory responses. Notably, although owing to the retrospective study design, the measurement of effectiveness of these therapies may have been biased by their close association with disease severity, owing to their selective use in clinical practice (e.g., corticosteroids are more commonly used in patients with the severe disease) [39], our findings still suggest a different response to immunotherapies therapy within different phenotypes. This needs to be considered in future trials.

### Limitations

Our study had several limitations. First, using 5 days as the cutoff point was somewhat arbitrary. Therefore, in the sensitivity analysis, we also assessed a trajectory model based on data within 3 days after admission (Additional file 1: Fig. S1), and the classification of phenotypes remained stable. Second, various inflammatory markers have been reported in COVID-19. However, only CRP levels, neutrophil counts, and lymphocyte counts were included as inflammatory markers in the current study, owing to limited data on other inflammatory markers. More studies are needed to further explore the relationship between other inflammatory markers, such as interleukins and tumor necrosis factor alpha, and temperature-trajectory phenotypes. Third, the association between immunotherapy and mortality in each temperature-trajectory phenotype may be biased owing to the greater likelihood of using these therapies in COVID-19 patients with more severe disease. However, the heterogeneous response to immunotherapy in patients with high and low inflammation phenotypes should be considered in future studies. Fourth, the number of patients with Phenotype 4 was relatively small. Therefore, a validation cohort was not used in the current study. Further studies are needed to verify our findings.

## Conclusion

We found that compared with normothermic patients and those with rapid fever defervescence, patients with gradual fever onset or slow defervescence temperature trajectories had a greater inflammatory response and higher mortality. There was a significant difference in response to immunotherapy among patients with different temperature-trajectory phenotypes. COVID-19 is a heterogeneous disease, and more studies are still needed to identify phenotypes with homogeneous features to improve the selection of medical interventions and the outcomes of COVID-19.

## Supplementary Information

**Additional file 1: Table S1.** Selection of different group-based temperature-trajectory phenotypes. **Fig. S1.** Four temperature trajectory phenotypes in COVID-19 patients based on daily maximum temperature data within 3 days. **Fig. S2.** Four trajectory phenotypes in COVID-19 patients without corticosteroid use within 5 days after hospital admission. **Fig. S3.** Four trajectory phenotypes in COVID-19 patients with hospital length of stay ≥ 5 days

## Data Availability

The full dataset is available from the corresponding author upon reasonable request.

## Abbreviations

ARDS: Acute respiratory distress syndrome
COVID-19: Coronavirus disease
CRP: C-reactive protein
SARS-CoV-2: Acute respiratory syndrome coronavirus 2
