# Supplementary material for: Novel phenotypes of coronavirus disease: a temperature-based trajectory model
Source: Ann Intensive Care. 2021 Aug 3;11:121. doi: 10.1186/s13613-021-00907-4 (PMC8330187; doi:10.1186/s13613-021-00907-4)
Supplement: Supplementary file 1 — Additional file 1: Table S1. Selection of different group-based temperature-trajectory phenotypes. Fig. S1. Four temperature trajectory phenotypes in COVID-19 patients based on daily maximum temperature data within 3 days. Fig. S2. Four trajectory phenotypes in COVID-19 patients without corticosteroid use within 5 days after hospital admission. Fig. S3. Four trajectory phenotypes in COVID-19 patients with hospital length of stay ≥ 5 days [file 13613_2021_907_MOESM1_ESM.docx]

**Additional file 1**

**Table S1** Selection of different group-based temperature-trajectory phenotypes

**Fig. S1** Four temperature trajectory phenotypes in COVID-19 patients based on daily maximum temperature data within 3 days.

**Fig. S2** Four trajectory phenotypes in COVID-19 patients without corticosteroid use within 5 days after hospital admission

**Fig. S3** Four trajectory phenotypes in COVID-19 patients with hospital length of stay ≥5 days

**Table S1 Selection of different group-based temperature-trajectory phenotypes**

| Group-based trajectory model | BIC | 2log_e_(B_10_) | AvePP | | | |
| --- | --- | --- | --- | --- | --- | --- |
|  |  |  | Phenotype-1 | Phenotype-2 | Phenotype-3 | Phenotype-4 |
| One trajectory group | −7799.8 | 14.4 | 1.0 ± 0.0  (n = 1580) |  |  |  |
| Two trajectory groups | −6879.7 | 12.2 | 0.98 ± 0.06 (n = 1330) | 0.93 ± 0.12 (n = 250) |  |  |
| Three trajectory groups | −6528.8 | 9.6 | 0.98 ± 0.06 (n = 1262) | 0.91 ± 0.14 (n = 189) | 0.93 ± 0.11 (n = 129) |  |
| Four trajectory groups | −6409.9 | Ref. | 0.97 ± 0.07 (n = 1217) | 0.92 ± 0.13 (n = 189) | 0.87 ± 0.15 (n = 122) | 0.93 ± 0.10 (n = 52) |

Abbreviations: BIC, Bayesian information criterion; AvePP, average posterior probability


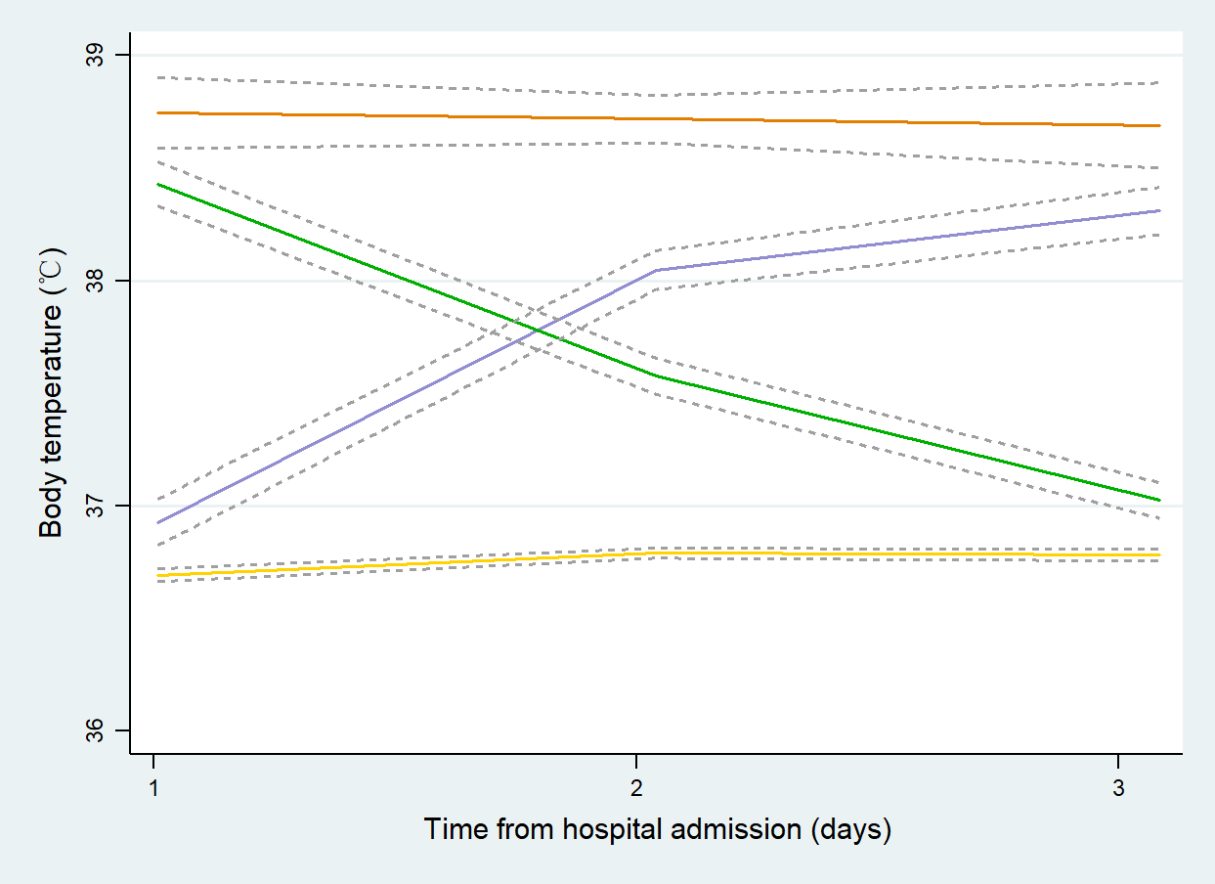


**Fig. S1** Four temperature trajectory phenotypes in COVID-19 patients based on daily maximum temperature data within 3 days.

Phenotype 1 (normothermic: yellow line) — patients with normal body temperature; Phenotype 2 (fever, rapid defervescence: green line) — patients with fever on admission but rapid defervescence; Phenotype 3 (gradual fever onset: purple line) — patients with normal temperature on admission who developed fever later; Phenotype 4 (fever, slow defervescence: orange line) — patients with fever on admission and slow defervescence. The dotted line represents the confidence interval.

COVID-19, coronavirus disease

**
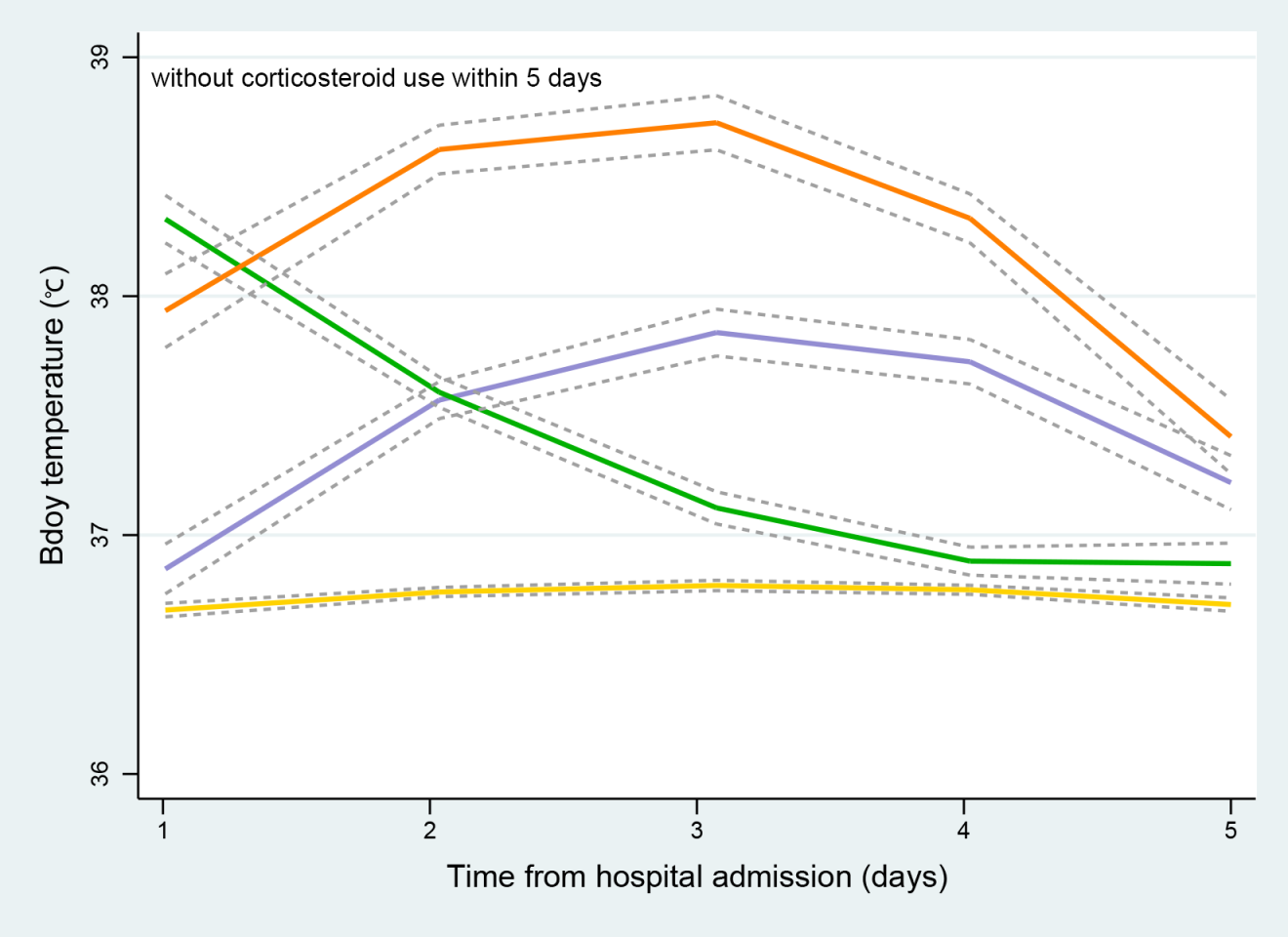
Fig. S2** Four trajectory phenotypes in COVID-19 patients without corticosteroid use within 5 days after hospital admission

Phenotype 1 (normothermic: yellow line) — patients with normal body temperature; Phenotype 2 (fever, rapid defervescence: green line) — patients with fever on admission but rapid defervescence; Phenotype 3 (gradual fever onset: purple line) — patients with a normal temperature on admission who developed fever later; Phenotype 4 (fever, slow defervescence: orange line) — patients with fever on admission and slow defervescence. The dotted line represents the confidence interval.

COVID-19, coronavirus disease

**
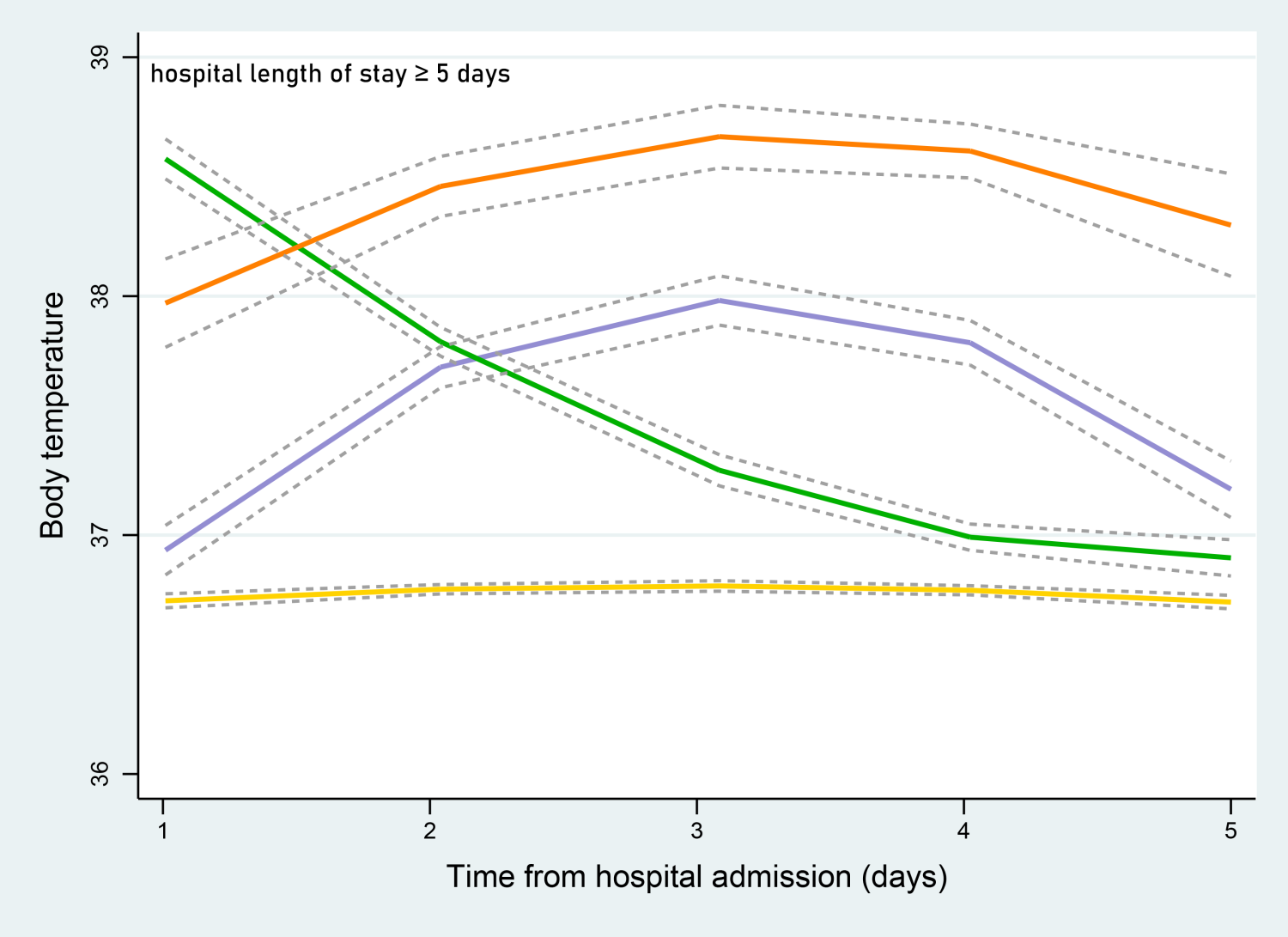
Fig. S3** Four trajectory phenotypes in COVID-19 patients with hospital length of stay ≥5 days

Phenotype 1 (normothermic: yellow line) — patients with normal body temperature; Phenotype 2 (fever, rapid defervescence: green line) — patients with fever on admission but rapid defervescence; Phenotype 3 (gradual fever onset: purple line) — patients with normal temperature on admission who developed fever later; Phenotype 4 (fever, slow defervescence: orange line) — patients with fever on admission and slow defervescence. The dotted line represents the confidence interval.

COVID-19, coronavirus disease


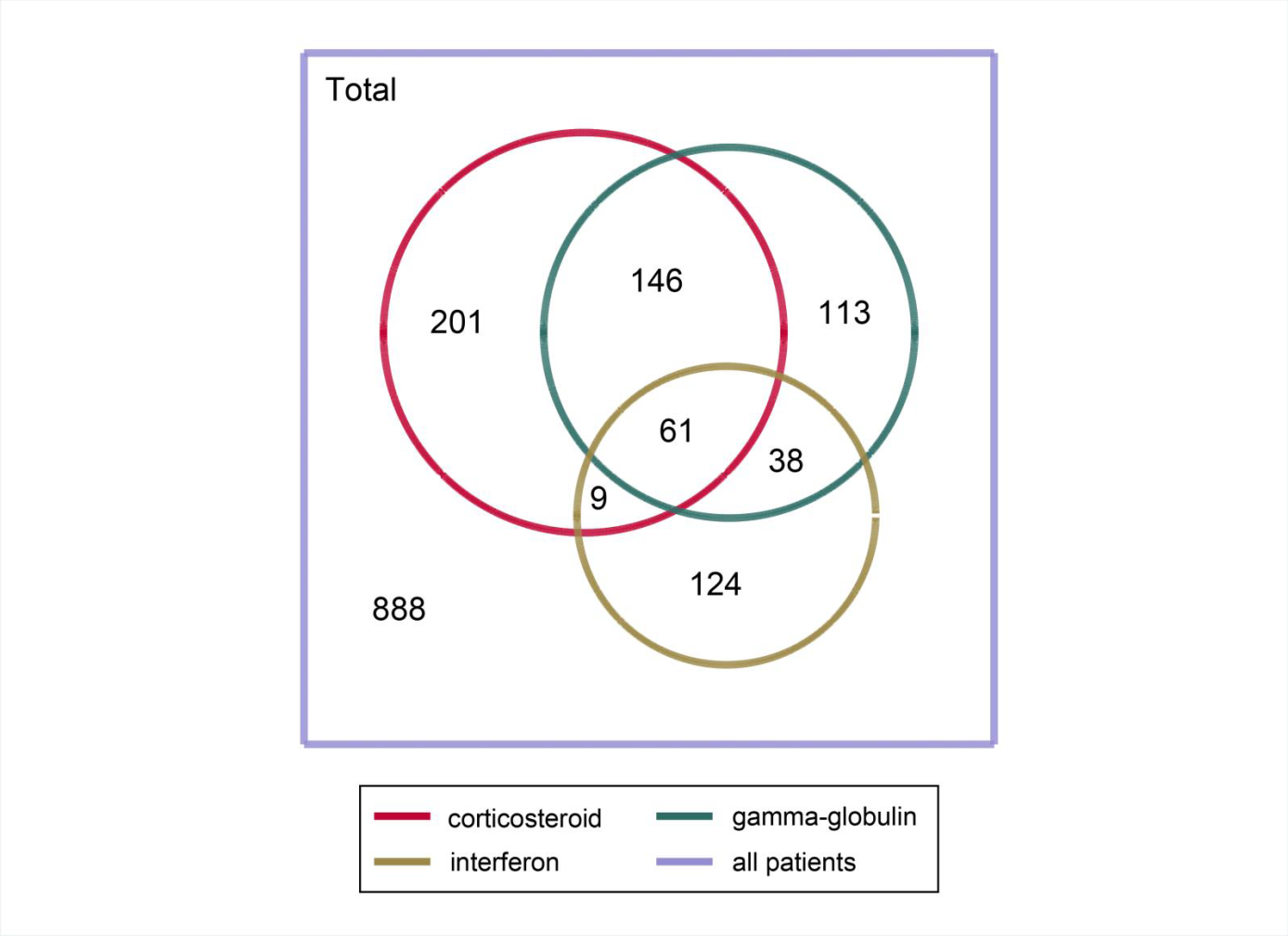


**Fig. S4** Venn diagram of the number of patients who received each immunotherapy

Note: The numbers within each area represent the number of patients who received that therapy combination. For example, 201 patients received only corticosteroid therapy.
